# Evaluation of lung toxicity with bevacizumab using the spontaneous reporting database

**DOI:** 10.1038/s41598-022-19887-x

**Published:** 2022-09-16

**Authors:** Yuko Kanbayashi, Mayako Uchida, Misui Kashiwagi, Hitomi Akiba, Tadashi Shimizu

**Affiliations:** 1 Department of Education and Research Center for Clinical Pharmacy, Faculty of Pharmacy, Osaka Medical and Pharmaceutical University, 4-20-1 Nasahara, Takatsuki, Osaka 569-1094 Japan; 2 Department of Education and Research Center for Pharmacy Practice, Faculty of Pharmaceutical Sciences, Doshisha Women’s College of Liberal Arts, 97-1, Kodominamihokotate, Kyotanabe-shi, Kyoto Japan; 3 School of Pharmacy, Hyogo Medical University, 1-3-6 Minatojima, Kobe, Hyogo Japan

**Keywords:** Cancer, Oncology, Medical research, Epidemiology

## Abstract

This study was undertaken to determine the risk of bevacizumab-induced lung toxicity, time to onset, and post hoc outcomes using the Japanese Adverse Drug Event Report database. We analysed data for the period between April 2004 and March 2021. Data on lung toxicities were extracted, and relative risk of adverse events (AEs) was estimated using the reporting odds ratio. We analysed 5,273,115 reports and identified 20,399 reports of AEs caused by bevacizumab. Of these, 1679 lung toxicities were reportedly associated with bevacizumab. Signals were detected for nine lung toxicities. A histogram of times to onset showed occurrence from 35 to 238 days, but some cases occurred even more than one year after the start of administration. Approximately 20% of AEs were thromboembolic events. Among these, pulmonary embolism was the most frequently reported and fatal cases were also reported. The AEs showing the highest fatality rates were pulmonary haemorrhage, pulmonary infarction, and pulmonary thrombosis. In conclusion, we focused on lung toxicities caused by bevacizumab as post-marketing AEs. Some cases could potentially result in serious outcomes, patients should be monitored for signs of onset of AEs not only at the start of administration, but also over a longer period of time.

## Introduction

Bevacizumab, a recombinant humanized monoclonal antibody against vascular endothelial growth factor (VEGF), inhibits tumour growth by blocking angiogenesis^1^. Combination chemotherapy with bevacizumab improves survival in patients with a variety of metastatic cancers^2–6^. In Japan, bevacizumab is approved for the treatment of advanced solid tumours, including colorectal cancer, non-small cell lung cancer, breast cancer, malignant glioma, ovarian cancer, cervical cancer, and hepatocellular carcinoma. Effective pharmacotherapy with bevacizumab requires appropriate management of adverse drug events that may occur with bevacizumab treatment. Inadequate management of adverse events (AEs) may necessitate discontinuation of bevacizumab therapy until the events are controlled, placing further burdens on patients such as decreased treatment efficacy. AEs reported with bevacizumab in post-marketing surveillance include hypertension, hemorrhage, proteinuria, and gastrointestinal perforation^7–9^. Hypertension is of particular interest^10^, and studies using the FDA Adverse Event Reporting System (FAERS) database have shown an increased risk of the occurrence of hypertension during bevacizumab administration^11^. In addition, studies using the Japanese Adverse Drug Event Reporting (JADER) database have shown an increased risk of developing thromboembolism, a serious adverse event^12^.

Conversely, VEGF inhibition has raised concerns about the occurrence of lung toxicities because VEGF is considered to play important roles in maintaining the survival of alveolar epithelial cells^13,14^. On the other hand, bevacizumab is a well-tolerated anti-tumour drug, its safety profile is well known, and AEs are manageable. Pulmonary hemorrhage associated with bevacizumab was reported as 2.1% and up to 8.9% in non-small cell lung cancer^15^. However, not only pulmonary haemorrhage, but also bevacizumab-induced lung toxicity such as interstitial lung disease, although less frequent, has been reported, and its onset can be fatal if not treated at an early stage^16^. Despite its potentially fatal onset, detailed AE information on bevacizumab-induced lung toxicity remains lacking^16–18^. We therefore aimed to clarify the risk of bevacizumab-induced lung toxicity, time to onset, and post hoc outcomes using the JADER database.

## Results

### Incidence of lung toxicities associated with bevacizumab

We joined the DRUG (3,875,874 reports), REAC (1,096,193 reports), and DEMO (693,295 patients) tables by ID number. We then removed duplicate data from the DRUG and REAC tables^19,20^. All AEs classified as “suspected drugs” were extracted totalling 5,273,115 reports that comprise the “data table”.

**Figure 1:**
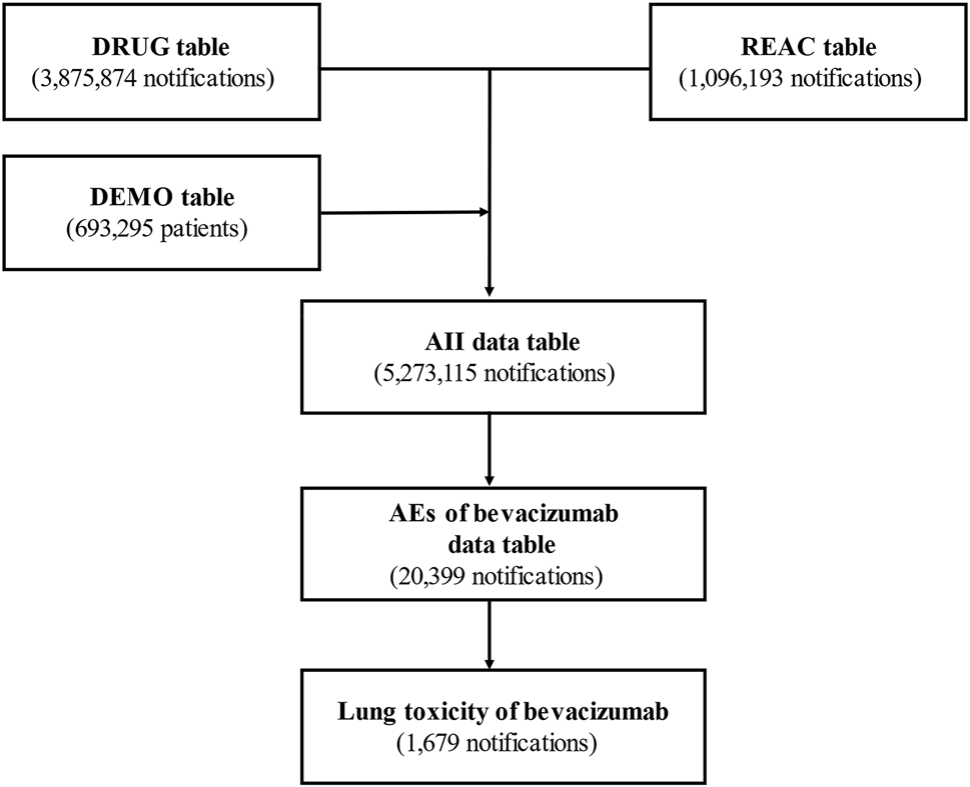
Process of constructing a data analysis table.

We analysed this data table and obtained 20,399 reports of AEs caused by bevacizumab. Of these, 1,679 lung toxicities were reportedly associated with bevacizumab (Fig. 1). The patient characteristics are shown in Table 1. Approximately 63.8% of patients were male. According to the age distribution of the study population, lung toxicity was more common among patients in their 70s (33.7%), followed by those in their 60s (34.8%).

**Table 1:** Characteristics of patients exhibiting lung toxicities related to bevacizumab.

Variable	Value (%)
Number of patients	1679
**Sex**	
Male	1071 (63.8)
Female	546 (32.5)
Unknown	62 (3.7)
**Age**	
10s	2 (0.1)
20s	1 (0.1)
30s	22 (1.3)
40s	84 (5.0)
50s	222 (13.2)
60s	585 (34.8)
70s	565 (33.7)
80s	94 (5.6)
90s	1 (0.1)
Unknown	103 (6.1)

Among the types of lung toxicity caused by bevacizumab, reported numbers of cases of interstitial lung disease, pneumonia, pulmonary embolism, pulmonary artery thrombosis, pneumonitis, lung disorder, respiratory failure, dyspnoea, cardio-respiratory arrest, and aspiration pneumonia associated with bevacizumab were 609, 246, 225, 86, 62, 54, 42, 37, 35, and 35, respectively (Table 2). Reporting odds ratios (ROR)s with a lower limit of the 95%CI > 1 comprised pulmonary embolism (5.28, 95%CI 4.61–6.05), pulmonary artery thrombosis (14.21, 95%CI 11.30–17.85), pneumonitis (1.85, 95%CI 1.44–2.38), lung disorder (1.37, 95%CI 1.05–1.79), pulmonary haemorrhage (1.97, 95%CI 1.30–2.97), pulmonary infarction (5.63, 95%CI 3.54–8.96), pulmonary thrombosis (8.14, 95%CI 5.02–13.21), pulmonary cavitation (8.24, 95%CI 3.79–17.89), and pulmonary venous thrombosis (13.42, 95%CI 5.23–34.45). Signals were thus detected for these nine lung toxicities with five or more cases reported.

**Table 2:** Numbers of reports and RORs of lung toxicities related to bevacizumab.

Variable	Cases (n)	Non-cases (n)	Rate (%)	ROR	95%CI	p-value
Interstitial lung disease	609	19,790	2.99	1.06	0.98–1.15	0.142
Pneumonia	246	20,153	1.21	0.87	0.76–0.98	**0.024**
Pulmonary embolism	225	20,174	1.10	5.28	4.61–6.05	** < 0.001**
Pulmonary artery thrombosis	86	20,313	0.42	14.21	11.30–17.85	** < 0.001**
Pneumonitis	62	20,337	0.30	1.85	1.44–2.38	** < 0.001**
Lung disorder	54	20,345	0.26	1.37	1.05–1.79	**0.025**
Respiratory failure	42	20,357	0.21	0.91	0.67–1.24	0.655
Dyspnoea	37	20,362	0.18	0.49	0.35–0.68	** < 0.001**
Aspiration pneumonia	35	20,364	0.17	1.08	0.77–1.51	0.658
Cardiorespiratory arrest	35	20,364	0.17	0.73	0.52–1.02	0.068
*Pneumocystis jirovecii* pneumonia	28	20,371	0.14	0.33	0.23–0.48	** < 0.001**
Acute respiratory distress syndrome	26	20,373	0.13	1.08	0.73–1.58	0.682
Pulmonary haemorrhage	23	20,376	0.11	1.97	1.30–2.97	**0.003**
Pulmonary alveolar haemorrhage	21	20,378	0.10	0.97	0.63–1.49	1.000
Pneumonia bacterial	21	20,378	0.10	0.59	0.38–0.90	**0.011**
Pulmonary infarction	19	20,380	0.09	5.63	3.54–8.96	** < 0.001**
Pulmonary thrombosis	18	20,381	0.09	8.14	5.02–13.21	** < 0.001**
Pulmonary tuberculosis	12	20,387	0.06	0.80	0.45–1.41	0.5161
Pulmonary oedema	11	20,388	0.05	0.38	0.21–0.68	** < 0.001**
Acute respiratory failure	10	20,389	0.05	0.84	0.45–1.57	0.769
Pulmonary hypertension	9	20,390	0.04	0.87	0.45–1.68	0.875
Eosinophilic pneumonia	8	20,391	0.04	0.47	0.23–0.94	**0.027**
Pulmonary cavitation	7	20,392	0.03	8.24	3.79–17.89	** < 0.001**
Pulmonary fibrosis	7	20,392	0.03	1.35	0.64–2.84	0.375
Lung abscess	6	20,393	0.03	2.05	0.91–4.60	0.080
Idiopathic pulmonary fibrosis	6	20,393	0.03	1.31	0.58–2.93	0.475
Organising pneumonia	6	20,393	0.03	0.34	0.15–0.75	**0.003**
Pulmonary venous thrombosis	5	20,394	0.02	13.42	5.23–34.45	** < 0.001**
Acute pulmonary oedema	5	20,394	0.02	2.03	0.83–4.92	0.107

“Cases” indicate the number of reported cases of pulmonary toxicity.

Bold p-values represent statistically significant results. We used more than five reports for each type of pulmonary toxicity. All analysed data were obtained from the Japanese Adverse Drug Event Report database. Hypothesis tests were two-sided, with statistical significance set at p < 0.05. P-values were calculated using Fisher’s exact test.

*ROR* reporting odds ratio, *95%CI* 95% confidence interval.

### Time to onset of lung toxicities associated with bevacizumab

**Figure 2:**
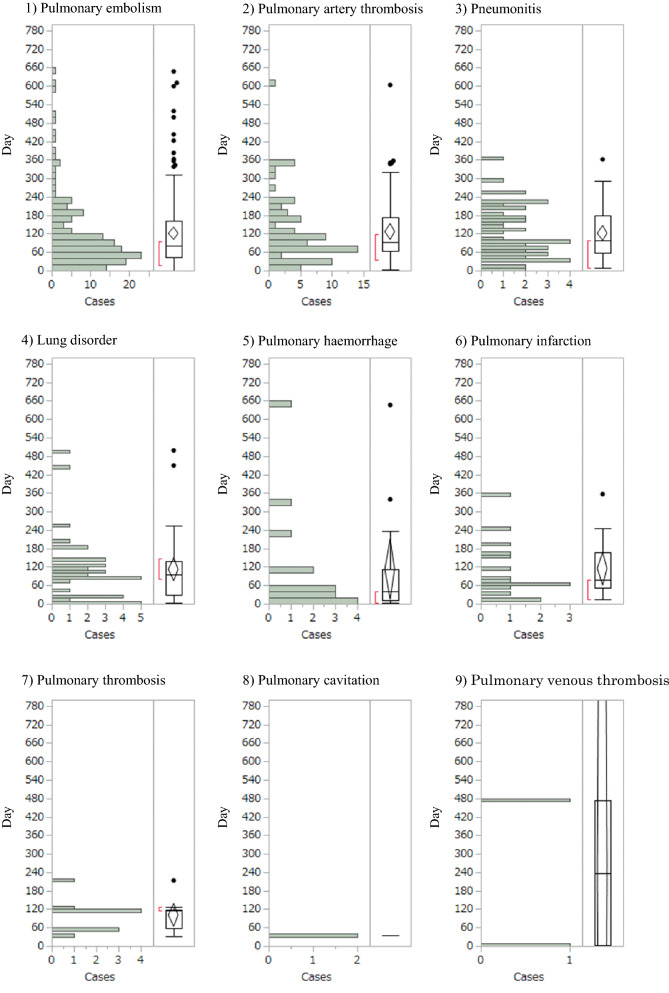
Histogram of lung toxicity for: (1) pulmonary embolism; (2) pulmonary artery thrombosis; (3) pneumonitis; (4) lung disorder; (5) pulmonary haemorrhage; (6) pulmonary infarction; (7) pulmonary thrombosis; (8) pulmonary cavitation; and (9) pulmonary venous thrombosis.

A histogram of times to onset for the nine detected lung toxicity signals showed occurrence from 35 to 238 days after bevacizumab administration (Fig. 2). Median times to onset were 81 days [interquartile range (IQR) 46–161 days] for pulmonary embolism, 93 days (IQR 64–170 days) for pulmonary artery thrombosis, 99 days (IQR 58–175 days) for pneumonitis, 96 days (IQR 29–135 days) for lung disorder, 42 days (IQR 12–113 days) for pulmonary haemorrhage, 78 days (IQR 53–168 days) for pulmonary infarction, 116 days (IQR 57–117 days) for pulmonary thrombosis, 35 days (IQR 35–35 days) for pulmonary cavitation, and 238 days (IQR 2–475 days) for pulmonary venous thrombosis caused by bevacizumab. The Weibull distribution of the histogram for time to onset showed that the ranges of 95%CIs for shape parameter β were < 1 for pulmonary haemorrhage and pulmonary venous thrombosis, approximately 1 for pulmonary embolism and lung disorder, and > 1 for the remaining 5 AEs (Table 3).

**Table 3:** Medians and Weibull parameters of lung toxicity.

Adverse effects	Case (n)	Median (days) (25–75%)	Scale parameter	Shape parameter
			α (95%CI)	β (95%CI)
Pulmonary embolism	149	81 (46–161)	123.91 (105.25–145.35)	1.06 (0.93–1.19)
Pulmonary artery thrombosis	73	93 (64–170)	137.00 (112.28–166.11)	1.25 (1.04–1.48)
Pneumonitis	45	99 (58–175)	134.23 (107.49–166.05)	1.45 (1.13–1.81)
Lung disorder	36	96 (29–135)	114.50 (81.35–158.83)	1.06 (0.80–1.34)
Pulmonary haemorrhage	15	42 (12–113)	83.30 (34.38–191.44)	0.67 (0.43–0.95)
Pulmonary infarction	15	78 (53–168)	123.98 (78.51–190.45)	1.29 (0.84–1.84)
Pulmonary thrombosis	10	116 (57–117)	113.97 (80.40–158.12)	2.15 (1.25–3.28)
Pulmonary cavitation	2	35 (35–35)	–	–
Pulmonary venous thrombosis	2	238 (2–475)	110.79 (0.20–86,174.68)	0.42 (0.09–1.13)

“Cases” indicate number of reported cases of pulmonary toxicity.

The detected pulmonary toxicity signals were analysed to determine time to onset.

*95%CI* 95% confidence interval.

### Outcomes after occurrence of AEs

**Figure 3:**
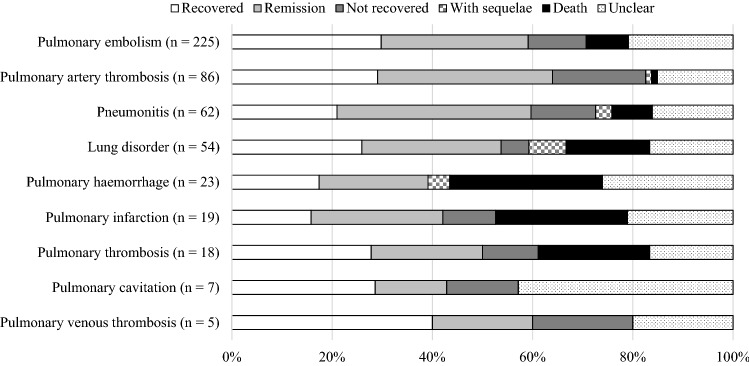
Percentage of nine AEs associated with bevacizumab by outcome.

Percentages of outcomes (recovery, remission, not recovered, with sequelae, death, and unclear) after onset of the nine AEs are shown in Fig. 3. Of the nine items for which signals were detected, fatal outcomes were observed in seven AEs. Among these, the AEs showing the highest fatality rates were pulmonary haemorrhage (30.4%), pulmonary infarction (26.3%), and pulmonary thrombosis (22.2%).

## Discussion

We focused on lung toxicities caused by bevacizumab, and the AEs for which signals were detected were pulmonary embolism, pulmonary artery thrombosis, pneumonitis, lung disorder, pulmonary haemorrhage, pulmonary infarction, pulmonary thrombosis, pulmonary cavitation, and pulmonary venous thrombosis.

We showed that approximately 20% of AEs (pulmonary embolism: 13.4% (225/1679); pulmonary artery thrombosis: 5.1% (86/1679); pulmonary infarction: 1.1% (19/1679); pulmonary thrombosis: 1.1% (18/1679); and pulmonary venous thrombosis: 0.3% (5/1679) = 21.0%) were thromboembolic events. Among these, pulmonary embolism was the most frequently reported and fatal cases were also reported. The Weibull distribution showed that the incidence of pulmonary embolism developed as a random failure-type AE. Pulmonary embolism associated to bevacizumab has been reported in several clinical trials^17,18,21,22^, and the present results are consistent with clinical findings. In a previous study, acute pulmonary thromboembolism shows a high mortality rate when untreated, and early diagnosis and treatment are known to significantly reduce mortality rates^23^. Pulmonary embolism and deep venous thrombosis are collectively termed venous thromboembolism (VTE), and pulmonary embolism represents the most dangerous type of VTE. Undiagnosed or untreated pulmonary embolism can be devastating and potentially fatal. Since pulmonary embolism is life-threatening and fatalities have been reported, clinicians need to continue careful monitoring during and after bevacizumab administration.

Of the nine items for which signals were detected, fatal outcomes were observed for seven AEs. Of these, pulmonary haemorrhage, pulmonary infarction, and pulmonary thrombosis exhibited high frequencies of fatalities. In this study, pulmonary haemorrhage is a fatal AE that occurs early after bevacizumab administration, a finding consistent with the results of clinical trials^24–26^. Clinicians thus need to be alert to the onset of symptoms of pulmonary haemorrhage right from the initial stages of bevacizumab treatment. On the other hand, although the incidence of pulmonary haemorrhage did not increase in a dose-dependent manner, continuous monitoring is recommended throughout and beyond the entire treatment period, as some cases of pulmonary haemorrhage were observed during the long term after the start of administration. The incidences of pulmonary infarction and pulmonary thrombosis increased in a dose-dependent manner. These AEs are also frequently reported as thromboembolism, consistent with previous studies^17,18,22^. In the same manner as for pulmonary haemorrhage, continuous monitoring throughout the entire administration period is recommended.

As for interstitial lung disease, a signal was detected in an analysis conducted by Kodama et al. using the JADER database from April 2004 to October 2017^27^. In our study using the JADER database from April 2004 to March 2021, no signal was detected for interstitial lung disease. Interstitial lung disease caused by bevacizumab is a rare AE. In addition, some reports have suggested that concomitant use of bevacizumab actually decreases the risk of interstitial pneumonia^28^. These reasons may have contributed to why no signal was detected for interstitial lung disease in this study.

In our study, most lung toxicities caused by bevacizumab occurred within 6 months after administration, but some cases of pulmonary embolism and pulmonary haemorrhage occurred even more than one year after the start of administration and proved fatal. Clinicians need to be aware of the possibility of such AEs developing after a long period of bevacizumab administration.

Among the nine AEs for which signals were detected, pulmonary embolism, pneumonitis and pulmonary haemorrhage are already listed in the technical data sheet in Japan. However, the detailed post-marketing data on the risk of pulmonary toxicity with bevacizumab, time to onset, and post-marketing results obtained in this study may be of great clinical significance for its safe and effective use.

The present study had some limitations that need to be considered. First, the JADER database is based on self-reports, which would introduce various reporting biases, including both over- and underreporting. Second, the lack of comprehensive medical records and medication histories limits the scope of the analysis, as dosages, durations, clinical laboratory data, severity of AEs and more information on concomitant medications of bevacizumab use were unavailable. Third, for the age group, the 70s (33.7%) and the 60s (34.8%) showed the majority. Thus, the higher age group cannot be ruled out as a causal factor in lung toxicity. Fourth, the possibility of AEs being caused by concomitantly used anticancer drugs cannot be ruled out. Fifth, potential confounding, selection, and information biases cannot be fully excluded in this study. However, the results of this study were based on extracted data in which bevacizumab was judged to be the suspect drug by the reporter (physician or pharmacist) who knew the details of the clinical course. Thus, our report provides useful information for monitoring lung toxicity AEs attributed to bevacizumab.

In conclusion, we focused on lung toxicities caused by bevacizumab as post-marketing AEs. Pulmonary embolism, pulmonary haemorrhage, pulmonary infarction, and pulmonary thrombosis could potentially result in serious outcomes after bevacizumab administration, and some cases have occurred even more than 1 year after starting administration. Patients should be monitored for signs of the onset of these AEs not only at the start of administration, but also over the long term.

## Methods

### Data source

Healthcare professionals and pharmaceutical companies send AE reports to the Pharmaceuticals and Medical Devices Agency (PMDA). Information from the JADER database^29–32^ was obtained from the PMDA website (https://www.pmda.go.jp/english/index.html, https://www.info.pmda.go.jp/fukusayoudb/CsvDownload.jsp) and includes AE cases. All data from the JADER database were fully anonymized by the regulatory authority before we accessed them. Ethics approval was not sought for this study, given the database-related, observational design without direct involvement of any research subjects. Thus, all methods were performed in accordance with the relevant guidelines and regulations. We analysed AE reports recorded between April 2004 and March 2021. The JADER consists of four datasets: patient demographic information (DEMO); drug information (DRUG); adverse events (REAC); and medical history (HIST). AEs in the JADER database were coded according to the terminology preferred by version 24.1 of the Medical Dictionary for Regulatory Activities/Japanese (https://www.pmrj.jp/jmo/php/indexj.php).

We first removed duplicate cases from the DRUG and REAC tables. We then used the identification number of each AE case to merge corresponding case data from the DRUG, REAC, and DEMO tables^19,20^. The contributions of medications to AEs were classified as “suspected drugs”, “concomitant drugs”, or “interaction”. Only cases classified as “suspected drugs” were extracted.

To investigate the association between bevacizumab and lung toxicities, we analysed the JADER database, which contains spontaneous AE reports submitted to the PMDA. We excluded missing data in our analysis.

### Statistical analyses

Data on lung toxicities with more than five reported cases were extracted, and the relative risk of AEs was estimated using ROR. ROR is frequently used in the spontaneous reporting database as an indicator of the relative risk of AEs. We used the analysis data table and constructed 2 × 2 tables based on two classifications: the presence or absence of “lung toxicity”; and the presence or absence of suspected bevacizumab use. RORs were calculated by dividing the reported rate of AEs attributable to bevacizumab by the reported rate of the same AEs attributable to all other drugs in the database. The signal from an AE was considered positive if the lower limit of the 95% confidence interval (95%CI) for the ROR was > 1^33^.

The time to onset of an AE was calculated and the number of cases was counted for reports in which the date of onset of the AE, the date of starting bevacizumab administration, and the date of ending bevacizumab administration were described as year/month/day or year/month^19,20^. Onset time was calculated as "(onset date of AE) − (start date of bevacizumab administration) + 0.5" in principle^34^. If a period of non-administration exceeding one year was identified, the date of first administration for the most recent continuous administration period was used. For analysis, the time to onset of AEs was limited to 2 years (730 days). The Weibull distribution is represented by scale parameter α and shape parameter β. Scale parameter α represents the scale of the distribution function, as the quantile in which 63.2% of AEs occur^35^. A large value of the scale indicates a wide distribution, while a small value indicates a narrow distribution. The shape parameter β represents a change in hazard over time in the absence of a reference population. Depending on the value of shape parameter β, an upper limit of the 95%CI for the β value < 1 indicates that the hazard initially increases, then decreases (early failure type), a β value of or almost 1 and a 95%CI including 1 indicates that the hazard remains constant throughout the exposure period (random failure type), and a lower limit of the 95%CI > 1 indicates that the hazard increases over time (wear-out failure type). All statistical analyses were performed using JMP Pro® version 16.1 (SAS Institute, Cary, NC, USA).

### Ethics approval

Ethics approval was not sought for this study, given the database-related, observational design without direct involvement of any research subjects. All results were obtained from data openly available online from the PMDA website (https://www.pmda.go.jp/english/index.html). All data from the JADER database were fully anonymized by the relevant regulatory authority before we accessed them. Thus, all methods were performed in accordance with the relevant guidelines and regulations.

## Data Availability

The datasets generated and analyzed during the current study are available from the corresponding author on reasonable request.
